# But, Who Is the Victim Here? Exploring Judgments Toward Hypothetical Bidirectional Domestic Violence Scenarios

**DOI:** 10.1177/0886260520917508

**Published:** 2020-05-12

**Authors:** Benjamin Hine, Ledja Noku, Elizabeth A. Bates, Kealey Jayes

**Affiliations:** 1 University of West London, UK; 2 University of Cumbria, Carlisle, UK

**Keywords:** intimate partner violence, bidirectional, mutual, interventions, attitudes

## Abstract

Gendered models of abuse describe intimate partner violence (IPV) as unilaterally perpetrated by dominant, aggressive men toward vulnerable women. This unidirectional conceptualization has contributed to a “domestic violence stereotype” which, alongside broader attitudes regarding gender, influences attitudes toward “non-typical” victim and perpetrator groups (e.g., male victims, female perpetrators, those within same-sex relationships), and has significant outcomes for help-seeking decision-making, as well as responses from service providers and the criminal justice system. While prevalence data and research suggest bidirectional violence is in fact the most common pattern, there is still little known about how the stereotypes and attitudes described above manifest in scenarios where both parties occupy “victim” and “perpetrator” labels. The present pilot study therefore asked 178 undergraduate students to allocate “victim” and “perpetrator” labels, and make judgments of severity, resolution, and justice outcomes, toward hypothetical opposite-sex IPV scenarios varying on the proportion of abuse perpetrated by each party, and type of violence. Results showed that participants were infrequently labelled men as “victims,” and women as “perpetrators,” across scenarios. They were also less likely to recommend that the man should call the police. These exploratory results suggest that powerful stereotypes about IPV and gender may serve to influence perceptions of bidirectional violence and point to a need to study this issue in more detail to elucidate the most appropriate way to begin to address these issues.

## Introduction

Much of the early research on intimate partner violence (IPV) was framed under a gendered, or feminist model (e.g., Dobash & Dobash, 1979). This is known as the “gender perspective” (Felson, 2002), and it posits that IPV is a problem of men’s violence toward women; specifically, that their physical aggression is part of a wider pattern of control and domination that has its roots in gender inequality and male privilege. It continues to be influential in policy and practice (e.g., Bates et al., 2017); indeed, popular programs of intervention based on this approach (e.g., the Duluth model; Pence & Paymar, 1993) frame IPV as unilaterally perpetrated by men, with attempts to address female violence labeled as victim blaming (Dutton & Corvo, 2007), or dismissed as solely motivated by self-defense (Dutton & Corvo, 2006). Notably, this “gender perspective” sees IPV as having a different etiology to other violence (Browne, 1987), meaning it should not be studied in the context of family violence, or other aggression.

Such conceptualizations of IPV have a significant influence on the attitudes of the general public, service providers, and those directly involved. For example, as the “gendered perspective” frames IPV as a harmful by-product of patriarchal society, and of a power inequality that permeates domestic relationships (Pagelow, 1984), male-perpetrated abuse toward a female victim garners more severe condemnation than any other gender combination (Ahmed et al., 2013; Arias & Johnson, 1989; Feather, 1996; Felson & Feld, 2009; Gerber, 1991; Harris & Cook, 1994; Hine, 2019; O’Toole & Webster, 1988; Poorman et al., 2003; E. P. Seelau et al., 2003; S. M. Seelau & Seelau, 2005; Willis et al., 1996). Such scenarios are also more likely to have police intervention recommended, are rated as more likely to be reported to the police, and are the most likely to receive a recommendation that the victim call the police. In contrast, women’s violence toward men is judged less harshly and as less likely to be illegal (Sorenson & Taylor, 2005). Indeed, in unpicking these hypothetical scenarios further, it is argued that gender, not the same- versus opposite-sex pairing, is most important as a predictor of these attitudes (E. P. Seelau et al., 2003; S. M. Seelau & Seelau, 2005).

Theoretical explanations for these findings have therefore tended to focus on gender-role stereotypes and the way we construct men and women in society. Indeed, both E. P. Seelau et al. (2003) and Hine (2019) argue that traditional gender-role stereotypes influence our perceptions of abuse. For example, as men are seen as powerful, self-reliant, and stoic (e.g., Vogel et al., 2011), they may be more readily identified as capable of abuse. Moreover, as women are purported to be vulnerable and dependent (e.g., Gerber, 1991), they are considered to be more in need of protection and are more readily identified as victims. Aggressive behavior is also seen as more synonymous with men’s gender roles, and as women have been shown to be less aggressive generally (see, for example, Archer, 2004), there is a tendency to seek to explain and attribute reason to their aggression (e.g., provocation; E. P. Seelau et al., 2003). Research further suggests that attributions made about male violence tend to point to an internal cause, whereas women’s violence was thought to be caused by external factors (Scarduzio et al., 2017), and that judgments about women’s aggression take more context-based factors into account (Sorenson & Taylor, 2005). As such, is has been suggested that powerful and pervasive norms exist around gender and IPV, which place strong, aggressive, powerful men as perpetrators of violence toward weak, vulnerable women (Hine, 2019).

Such stereotypes have a considerable impact on victim experience. For example, the status of “victim” does not appear to carry the same credibility for men as for women (E. P. Seelau et al., 2003), thus affecting the ability of men to recognize and label their experiences of abuse (Machado et al., 2016). Moreover, personal and external reactions to experiences of IPV influence help-seeking decisions, as well as support received (Hine, 2019). Indeed, Bates (2019b) found that in her non-help-seeking sample, men described these perceptions as leaving them feeling “weak,” being perceived as the abuser, and not identifying as a “victim” of IPV. This was then often cited within their narratives as reasons they had chosen not to seek formal help from services or the police, and other studies support the assertion that men often do not report their experience for fear of not being taken seriously (Drijber et al., 2013). This is further supported by research which, unsurprisingly, shows that men are blamed more for their victimization by the general population (Taylor & Sorenson, 2005) and that such judgments are reflected by service professionals (e.g., police officers; McCarrick et al., 2016; Stewart & Maddren, 1997).

Around the same time that the gendered model was developing, a parallel and contrasting body of work that saw IPV as one form of family violence also emerged, and indeed understood it within this wider context. This is what Felson (2002) labelled the “violence perspective.” Such an approach seeks to understand violence and it’s characteristics on an individual level, rather than seeing IPV as a societal issue requiring social change, and utilizes more representative samples and methods, including gender neutral, self-report surveys (e.g., Conflict Tactics Scale, CTS; Straus, 1979). This approach has been critical in revealing that women can be as abusive as men in relationships (Archer, 2000), that women are more controlling than men (Bates & Graham-Kevan, 2016), and that IPV, general aggression, and control are all significantly related, regardless of gender (e.g., Bates et al., 2014). Indeed, these findings have generated a more in-depth exploration of both women’s aggression (e.g., Mackay et al., 2018) and men’s victimization (e.g., Drijber et al., 2013; Hines et al., 2007). For example, quite in contrast to earlier hypotheses, both men and women cite power and control, as well as self-defense, as the most common motivations for IPV perpetration, with expressions of negative emotion and jealousy common, alongside communication difficulties (see Langhinrichsen-Rohling, McCullars, & Misra, 2012, for review). Similarly, where women’s violence was previously asserted to be trivial and not impactful, more recent literature details the severity of such acts, in terms of both physical and psychological harm (e.g., Bates, 2019b; Hines & Douglas, 2010, 2011) Thus, while the gendered model is still dominant, there is now a wealth of evidence that indicates IPV is far more complex than this framework allows.

Importantly, through the use of large-scale, gender neutral surveys, the “violence perspective” has strongly challenged the idea that IPV is principally unidirectional, revealing instead substantial similarity between men’s and women’s perpetration of IPV, as well as the prevalence of bidirectional or mutual IPV. For example, Straus (2008) found in a sample of more than 13,000 male and female students across 32 nations that the most frequent pattern of abuse is bidirectional, followed by female-only perpetration, with male-only perpetration least frequently reported. Moreover, in a comprehensive literature review of 48 studies reporting rates of bidirectional versus unidirectional IPV, Langhinrichsen-Rohling, Misra, et al., (2012) calculated a weighted rate of violence across the collated studies (*N* = 2,991; 1,615 women and 1,376 men), which showed prevalence of violence across these samples was 47.0%, and of this, 59.6% was bidirectional violence. The remaining 40.4% was unidirectional which was further categorized into 17.5% male to female and 22.9% female to male. Such results suggest that current conceptualizations of IPV as solely male-perpetrated and the associated stereotypes, while widespread, are fundamentally inaccurate.

While the existing literature has contributed to our understanding of how gender affects perceptions of IPV, as well as how these perceptions impact on help-seeking and reporting, there is a dearth of research exploring the manifestation of such attitudes and attributions in scenarios involving bidirectional IPV. Such investigation is critical when considering the prevalence of this type of IPV within relationships, as well as the severity of such abuse, both in terms of likelihood of injury and mental health problems (e.g., Whitaker et al., 2007), including post-traumatic stress disorder (PTSD) symptoms, depression, and suicidality (Rhodes et al., 2009). Moreover, much of the literature on IPV, including that concerning attitudes, theory, treatment, and prevention, is consistent in its use of the dichotomous terms *perpetrator* and *victim*. While this is clearly appropriate for unidirectional IPV, exploring the allocation of such terms within bidirectional scenarios is necessary in understanding how such incidents are interpreted when those involved occupy both categories (Bates, 2016), as well as the impact of additional factors (e.g., who initiates the incident; Dutton & Corvo, 2006). Similarly, there has been a historic tendency within the IPV literature to focus on the physical aggression, at the cost of a lesser understanding of other more prevalent forms of abuse (Straight et al., 2003). While this has recently begun to change within the literature (see review by Carney & Barner, 2012), there is still a dearth of knowledge about the perceptions of this type of aggression. The present study therefore sought to assess how judgments of IPV in bidirectional scenarios differ as a function of proportion of abuse perpetrated by each partner, the initiator of the aggression, and type of abuse. Three research questions were proposed for this exploratory pilot study:

**Research Question 1:** Do participants’ judgments of the individuals involved in bidirectional domestic disputes vary based on the proportion of abuse, type of abuse, or abuse initiator, and the interactions between them?
**Research Question 2:** Do participants’ judgments of the situation (e.g., seriousness) vary based on the three factors named above?
**Research Question 3:** And do participants’ suggestions for resolution and outcome vary based on the three factors named above?

## Method

### Design

This study adopted a between-subjects design with three factors: abuse type (with two levels: physical and psychological/emotional), proportion of abuse perpetration (with three levels: male-dominated, equal, and female-dominated), and initiator (with two levels: male-initiated and female-initiated). These factors constituted the independent variables in this study. Two additional variables of interest, previous experience of an abusive relationship (with two levels, yes and no) and participant gender (with two levels, male and female), were preliminarily included in some analyses, but were eliminated as variables of interest when no significant differences were found between those who did and did not report previous experience, or between men and women. Twelve questions measuring perceptions of the scenario and those involved, allocation of perpetrator/victim labels, and suggested actions and outcomes, acted as the dependent variables in this study.

### Participants

A total of 178 undergraduate students (*M*_age_= 26.03 years, *SD* = 8.56, 116 women) took part in this study in 2018. Participants were recruited campus-wide from a university in the South of England and identified as being from a variety of ethnic backgrounds (44% White British, 23% Other White Background, 33% non-White Ethnic Background). Importantly, for this study, 28 participants (16%) said that they had experienced some form of IPV in the past (with 6% preferring not to say), although whether this was from the perspective of victimization or perpetration was not asked (as the question was considered unnecessarily invasive, and difficult to operationalize when acknowledging the frequency of bidirectional abuse). No incentives were offered for participation.

### Materials

A vignette depicting an incident of IPV was created using examples from previous research (Hine, 2019; S. M. Seelau & Seelau, 2005). Scenarios detailed a one-time incident of violence within an opposite-sex couple. Variations in the proportion of perpetrated abuse were created by allocating a different number of violent acts to the male or female in the scenario. In “male-dominated” scenarios, the man in the scenario perpetrated three acts of violence to one act by the woman, with the opposite true for “female-dominated scenarios.” In the “equal” scenario, each person committed three acts of violence. To verify that acts of abuse were easily identifiable, five independent judges (departmental colleagues) were asked to highlight incidents of abuse within each scenario. All violent acts were correctly identified. Abuse type was also varied by directly interchanging acts of physical versus psychological violence (in line with definitions provided by the charity *Safelives*). Finally, who initiated the interaction was varied by having either the male or the female act first within the scenario. Example vignettes, showing differences across factors, are included below (with acts of abuse in highlighted in bold, and abuse sequence and gender of perpetrator given in superscript):

Female Dominated, Physical Abuse, Female Initiator:

Please read the following scenario involving an account of conflict between a romantic couple. Mark, a 27-year old male, and Kelly, a 26-year old female. Mark and Kelly have been together for approximately three years. They both weigh roughly 12.5 stone and are 5 foot 10 inches tall.

Kelly returns home from work one Friday evening after doing overtime. She has a deadline to meet on Monday so staying back was the only way of being able to get the work done. This was unplanned and had not been mentioned to Mark. As she enters the door, Mark begins to question her on her whereabouts, asking whether she has been with another man. **Kelly slaps Mark across the face**^1F^, saying he is being silly, that she simply had a load of work to get done and she is tired and wants to go to sleep. As the argument escalates, Kelly walks out of the sitting room and heads towards the bedroom. **Mark grabs her by the arm hard enough to leave a mark**^1M^. She pulls her arm away from him, **scratching Mark’s arm with her other hand**^2F^. She looks at Mark and tells him she has not, and is not, cheating before **picking up a nearby glass and throwing it at Mark**^3F^. Kelly walks to the bedroom, shuts the door and stays in the bedroom; Mark stays in the sitting room.

Male Dominated, Psychological Abuse, Male Initiator:

Please read the following scenario involving an account of conflict between a romantic couple. Mark, a 27-year old male, and Kelly, a 26-year old female. Mark and Kelly have been together for approximately three years. They both weigh roughly 12.5 stone and are 5 foot 10 inches tall.

Kelly returns home from work one Friday evening after doing overtime. She has a deadline to meet on Monday so staying back was the only way of being able to get the work done. This was unplanned and had not been mentioned to Mark. As she enters the door, Mark begins to question her on her whereabouts, **insisting she has been with another man, wanting to know who else stayed back late at work**^1M^. Kelly says Mark is being silly, that she simply had a load of work to get done and she is tired and wants to go to sleep. Mark tells Kelly that she prioritises work over their relationship. As the argument escalates, Kelly walks out of the sitting room and heads towards the bedroom. **Mark says he’s going to call some of her colleagues to see if she’s telling the truth**^2M^, Kelly ignores him. **Mark calls her a “Bitch!” and tells Kelly she is ‘fat and ugly’**^3M^. Kelly sighs, looks back and tells him she has not, and is not cheating. As she goes to close the door she says “**would you really be surprised if I did though, I might get to be with a real man for a change”**^1F^. *Kelly shuts the door and stays in the bedroom; Mark stays in the sitting room.*

Participants then answered 12 questions about the scenario and those involved. Five questions concerned perceptions of the individuals involved in the scenario (e.g., “Who do you believe is/are the victim(s) in this dispute?” and “How responsible is Mark for this situation?”); three questions asked about perceptions of the incident itself (e.g., “How serious do you consider this case to be?”); and four questions asked about the outcome and/or resolution of the incident (e.g., “Had you witnessed this conflict, what would you have done?”). Some questions (e.g., How serious do you consider this scenario to be?) involved answering on a Likert-type scale from 1 (*not at all*) to 5 (*extremely*). Other questions (e.g., who is/are the perpetrator(s) in this dispute?) invited categorical responses (e.g., Mark, Kelly, or Both).

### Procedure

Potential participants were approached in quiet spaces around campus (i.e., library, study spaces) and invited to read an information sheet about the study. If they were interested in taking part, they were then asked to provide informed consent. An electronic version of the questionnaire battery was then presented to participants using the survey software Qualtrics, either on a tablet used by the researcher or an electronic device owned by the participant. They were first asked demographic questions, before being randomly assigned one of the 12 different IPV scenarios (participants only saw one scenario). Written text asked participants to read this carefully, and to answer the questions presented honestly. Once participants were finished, they were presented with debriefing information (both electronically and in paper form), including contact information for local support services if required. This study was approved by the departmental ethics boards of the first and second authors’ institutions.

## Results

Data from questions inviting categorical responses were subjected to chi-square analysis. Each independent variable (type of abuse, proportion of abuse perpetrated, abuse initiator, and participant gender) was assessed separately, as sample numbers in this study did not support the layering of cross tabs (i.e., too many cells contained values of less than 5), and values of zero in some cells prohibited multinomial logistic regression. It is important to note that, while it is possible to establish whether values are unevenly distributed across cells, it is not possible to formally use post hoc analyses to establish where specific differences lie between individual cells. Therefore, when chi-square analysis was significant, the authors interpretation of the values responsible for this significance are highlighted in bold in the appropriate tables. 2 (Type of Abuse) × 3 (Proportion of Abuse Perpetrated) × 2 (Abuse Initiator) analyses of variance (ANOVAs) were conducted on data from all other questions. Levene’s test for equality of variances was conducted as part of all ANOVA and was significant for all tests. However, as group sizes were roughly equal across conditions, this means results can still be reliably interpreted, although with some caution.

### Judgment of Individuals

Chi-square analysis revealed a significant influence of proportion of abuse perpetrated on who participants allocated as the victim within their scenario, χ^2^(4) = 25.44, *p* < .001. Specifically, results showed that, while a consistent number of participants said that “both” Mark and Kelly were the victim, participants frequently labeled Kelly as the primary victim in both the “male dominated” and “equal” scenarios (see Table 1). Interestingly, in the “female dominated” scenario, only one less person labeled Kelly as the sole victim than Mark (10 vs. 11, respectively). Such results suggest that, while participants appeared to acknowledge that both parties had aggression perpetrated against them, they infrequently labeled men as the primary victims, even when they suffered the majority of abuse.

**Table 1.** Cell Counts (and Percentages) for Participants’ Choices of Victim, Perpetrator and Who Is in the Wrong Across Proportion of Abuse Conditions.

Question	Kelly	Both	Mark
Who is the victim?			
Male dominated	29 (45.3)	33 (51.6)	**2 (3.1)**
Equal	22 (37.3)	37 (62.7)	**0 (0.0)**
Female dominated	10 (18.5)	33 (61.1)	**11 (20.4)**
Who is the perpetrator?			
Male dominated	**2 (3.1)**	24 (37.5)	38 (59.4)
Equal	**4 (6.8)**	23 (39.0)	32 (54.2)
Female dominated	**14 (26.4)**	23 (43.4)	16 (30.2)
Who is in the wrong?			
Male dominated	**0 (0.0)**	31 (49.2)	32 (50.8)
Equal	**2 (3.4)**	38 (64.4)	19 (32.2)
Female dominated	**7 (13.0)**	36 (66.7)	11 (20.4)

*Note.* Values in bold highlight extreme values of interest where chi-square tests have been significant.

A significant influence of proportion of abuse perpetrated was also found for participants allocation of the perpetrator label, χ^2^(4) = 21.27, *p* < .001. Specifically, results revealed that in “male dominated” and “equal” scenarios, participants were more likely to label Mark as the sole perpetrator than Both or Kelly (see Table 1). Moreover, in “female dominated” scenarios, two more participants labeled Mark as the sole perpetrator than Kelly, despite the fact that Kelly is perpetrating the majority of violence. This suggests that participants may be overly willing to label men as perpetrators, even in scenarios where they are perpetrating an equal share of abuse or have only provided one abusive action. Similar results were found when assessing the influence of proportion of abuse on who participants labeled as being “in the wrong,” χ^2^(4) = 19.76, *p* < .001. In that, participants were much more likely to say that Mark was in the wrong in “male dominated” and “equal” scenarios than Both or Kelly. Again, even in “female dominated” scenarios, more participants said Mark was in the wrong than Kelly (although more participants chose Both than either of these options, see Table 1).

Type of abuse also had a significant effect on judgments of who was the victim, χ^2^(4) = 6.09, *p* < .05, and who was the perpetrator, χ^2^(4) = 7.39, *p* < .05; see Table 2. Moreover, abuse initiator had a significant effect on judgments of who was the victim, χ^2^(4) = 7.03, *p* < .05, and who is in the wrong, χ^2^(4) = 13.27, *p* < .001; see Table 3. However, the patterns followed those outlined above, with participants infrequently labeling Mark as the victim, or Kelly as the perpetrator/in the wrong across all conditions. Such results suggest that differences in conditions manipulated within this study failed to have an influence on participants allocations. Instead, participants’ judgments were perhaps underpinned by well-known stereotypes regarding abuse; that it is perpetrated by men, toward women.

**Table 2.** Cell Counts (and Percentages) for Participants’ Choices of Victim, Perpetrator and Who Is in the Wrong Across Type of Abuse Conditions.

Question	Kelly	Both	Mark
Who is the victim?			
Physical	24 (26.1)	61 (66.3)	**7 (7.6)**
Psychological	37 (43.5)	42 (49.4)	**6 (7.1)**
Who is the perpetrator?			
Physical	**13 (14.1)**	43 (46.7)	36 (39.1)
Psychological	**7 (8.3)**	27 (32.1)	50 (59.5)
Who is in the wrong?			
Physical	5 (5.4)	59 (64.1)	28 (30.4)
Psychological	4 (4.8)	46 (54.8)	34 (40.5)

*Note.* Values in bold highlight extreme values of interest where chi-square tests have been significant.

**Table 3.** Cell Counts (and Percentages) for Participants’ Choices of Victim, Perpetrator and Who Is in the Wrong Across Initiator conditions.

Question	Kelly	Both	Mark
Who is the victim?			
Male initiator	35 (43.8)	42 (52.5)	**3 (3.8)**
Female initiator	26 (26.8)	61 (56.4)	**10 (10.3)**
Who is the perpetrator?			
Male initiator	6 (7.5)	28 (35.0)	46 (57.5)
Female initiator	14 (14.6)	42 (43.8)	40 (41.7)
Who is in the wrong?			
Male initiator	**5 (6.3)**	36 (45.0)	39 (48.8)
Female initiator	**4 (4.2)**	69 (71.9)	23 (24.0)

*Note.* Values in bold highlight extreme values of interest where chi-square tests have been significant.

A 2 × 3 × 2 ANOVA revealed that proportion of abuse also had a significant effect on responsibility judgments for Mark, *F*(2, 165) = 3.30, *p* < .05, with him judged as significantly less responsible in female-dominated scenarios than male-dominated and equal scenarios (see Table 4). No other main effects or interactions for Mark’s responsibility were found; however, the findings above were complemented by results showing that Kelly was judged as significantly less responsible in male-dominated scenarios than female-dominated and equal scenarios, *F*(2, 165) = 3.43, *p* < .05. Type of abuse also had a significant main effect on judgments of Kelly’s responsibility, *F*(1, 165) = 11.58, *p* < .001, with her judged as more responsible in physical versus psychological scenarios. Such results may speak to participants’ negative judgment of physical violence by women, as it violates gender-role expectations. A significant main effect was also found for abuse initiator, *F*(1, 165) = 11.58, *p* < .001, with Kelly judged as more responsible when she initiated the abuse than when she did not. Interestingly, an interaction effect was found between type of abuse and abuse initiator, *F*(2, 165) = 10.05, *p* < .01. Post hoc *t* tests revealed that, in psychological scenarios, no significant differences were found for responsibility judgments between male-initiated (*M* = 2.33, *SD* = 1.16) and female-initiated scenarios (*M* = 2.52, *SD* = 1.07). However, in physical abuse scenarios, Kelly was judged as more responsible when she initiated the abuse (*M* = 3.64, *SD* = 1.16) than when she did not, *M* = 2.36, *SD* = 1.23, *t* (90) = 5.16, *p* < .001.

**Table 4.** Means (Standard Deviations) for Participants’ Judgments of Responsibility for Mark and Kelly, Incident Seriousness, and Severity of Mark and Kelly’s Injuries Across All Three Factors (Only the Relevant Factor of Proportion of Abuse Was Assessed for Judgments of Injury).

Condition	Mark’s Responsibility	Kelly’s Responsibility	Seriousness	Mark’s Injuries	Kelly’s Injuries
Proportion of abuse					
Male dominated	3.68_a_ (1.09)	2.43_c_ (1.24)	3.23 (1.17)	2.40 (1.15)	2.92_k_ (1.18)
Equal	3.65_a_ (1.06)	2.88_d_ (1.24)	3.20 (1.16)	2.39 (1.13)	2.76_k_ (1.18)
Female dominated	3.17_b_ (1.24)	2.96_d_ (1.27)	2.83 (1.14)	2.67 (1.18)	2.17_l_ (1.09)
Abuse Type					
Physical	3.62 (1.09)	3.03_e_ (1.34)	3.53 (1.11)_i_	2.63 (1.22)	2.73 (1.23)
Psychological	3.41 (1.19)	2.44_f_ (1.11)	2.64 (1.03)_j_	2.31 (1.06)	2.55 (1.15)
Abuse initiator					
Male initiated	3.76 (1.10)	2.35_g_ (1.19)	3.07 (1.13)	2.44 (1.11)	2.63 (1.18)
Female imitated	3.49 (1.08)	3.08_h_ (1.24)	3.13 (1.19)	2.51 (1.19)	2.66 (1.21)

*Note.* Values with different letters indicate significant differences to *p* < .05.

### Situational Judgments

A 2 × 3 × 2 ANOVA was conducted to assess the influence of proportion of abuse perpetrated, abuse type, and abuse initiator on participants’ judgments of how serious they considered the scenario to be (see Table 4). Results showed a marginal main effect of proportion of abuse perpetrated, *F*(2, 166) = 2.63, *p* = .07, with results showing a trend toward participants judging “female-dominated” scenarios as less serious than both “male-dominated” and “equal” scenarios. This suggests that participants may consider women to be less threatening as perpetrators, with their acts of aggression classed as less severe. A main effect was also found for type of abuse, *F*(1, 166) = 30.45, *p* < .001, with participants judging physical abuse as significantly more serious than psychological abuse. This may speak to participants’ perceptions of physical aggression as more damaging and apparent than psychological aggression.

Two one-way ANOVAs were also conducted to assess the influence of proportion of abuse perpetration on participants’ judgments of injury severity for Kelly and Mark separately (see Table 4). No significant results were found for Mark, as participants judged his injuries similarly across all conditions. Conversely, participants judged Kelly’s injuries to be more serious in “male-dominated” (*M* = 2.92, *SD* = 1.18) and “equal” scenarios (*M* = 2.76, *SD* = 1.18) than in “female-dominated” scenarios, *M* = 2.17, *SD* = 1.09, *F* (2, 174) = 6.60, *p* < .01.

### Outcome and Resolution

Six chi-square analyses were conducted to assess the influence of proportion of abuse perpetration, abuse type, and abuse initiator on participants’ judgments of how to resolve the dispute, and participant’s choices of intervention. Significant differences were only found for type of abuse on witness reaction, χ^2^(3) = 17.74, *p* < .001, with participants more likely to recommend doing nothing, and less likely to recommend calling the police in psychological versus physical violence (see Table 5). No significant differences were found for any other analyses, with most participants indicating that the couple should try to talk things through alone, and that they would try and talk to the couple across conditions.

**Table 5.** Cell Counts (and Percentages) for Participants’ Choices of What They Would Do If They Had Witnesses the Conflict Across Type of Abuse Conditions.

Condition	Nothing	Try to talk to couple	Call a hotline/organization	Call the police
Abuse type				
Physical	**6 (6.5)**	56 (60.2)	7 (7.5)	**24 (25.8)**
Psychological	**17 (20.0)**	58 (68.2)	0 (0.0)	**10 (11.8)**

*Note.* Values in bold highlight extreme values of interest where chi-square tests have been significant.

Two further chi-square analyses were conducted to assess the influence of proportion of abuse on participants’ judgments of who should call the police and who should press charges. Results revealed a significant effect on participants choices of who should call the police, χ^2^(4) = 19.64, *p* < .001, and who should press charges, χ^2^(4) = 25.97, *p*< .001, as a greater number of participants said that Kelly should call the police and press charges in the “male-dominated” and “equal” scenarios, with roughly equal amounts of participants choosing Mark, Kelly, and Both in the “female-dominated scenario” (see Table 6). Taken together, such results suggest that participants largely view domestic abuse as something to be resolved without legal action, and that women rather than men should seek judicial support.

**Table 6.** Cell Counts (and Percentages) for Participants’ Choices for Who Should Call the Police Across Proportion of Abuse Perpetration Conditions.

Question	Kelly	Both	Mark
Who should call the police?			
Male dominated	38 (65.5)	17 (29.3)	**3 (5.2)**
Equal	21 (44.7)	22 (46.8)	**4 (8.5)**
Female dominated	**15 (33.3)**	**17 (37.8)**	**13 (28.9)**
Who should press charges?			
Male dominated	35 (61.4)	21 (36.8)	**1 (1.8)**
Equal	20 (42.6)	24 (20.3)	**3 (6.4)**
Female dominated	**12 (27.3)**	**19 (43.2)**	**13 (29.5)**

*Note.* Values in bold highlight extreme values of interest where chi-square tests have been significant.

## Discussion

This study examined the influence of proportion of perpetration, as well as abuse type and abuse initiator, on the allocation of “victim” and “perpetrator” labels, and judgments of severity, resolution options, and justice outcomes in an IPV scenario. This is the first study to examine the influence of these factors in the context of mutual or bidirectional IPV, where participants in the incident occupy both victim and perpetrator roles. Results indicated that, while a significant proportion of participants recognized the bidirectional nature of the abuse, many appeared to be influenced by the same beliefs that inform judgments of unidirectional abuse (as shown in previous research). Such results speak to the pervasiveness of the gendered stereotypes and attitudes surrounding IPV, perpetuated by the “gender perspective.” Importantly, such results suggest that, even when abuse is clearly bidirectional, we may be reluctant to see men as victims, and women as perpetrators, of abuse.

In reference to "victim" and "perpetrator" labels, it can be argued that participants should allocate labels in one of two ways. First, participants could provide the label of both for all scenarios, as technically all incidences include perpetration and victimization for each party. Alternatively, participants could allocate labels broadly in line with the proportion of abuse (i.e., more female victim allocations in the male-dominated scenario and vice versa). However, results demonstrate that neither occurred. Instead, participants infrequently labeled women as primary perpetrators or as “in the wrong,” even in circumstances where they were shown to be perpetrating the majority of the aggressive acts. Indeed, even in “female-dominated” scenarios, the man was marginally more likely to be labeled as the abuser than the woman. Opposite patterns were shown for victim label allocations, as participants infrequently gave men this label, even when the violence was predominantly female perpetrated. The results were mirrored when assessing for the influence of abuse initiator, as regardless of whether the man or woman was violent first, participants were still more likely to label Kelly as the victim and Mark as the perpetrator. Such results serve to undermine ideas around female violence as occurring solely as self-defense (Dutton & Corvo, 2006), but instead speak to a much broader dismissal of female aggression, and male victimization.

Taken together, these findings suggest that traditional stereotypes which portray men as inherently aggressive (Vogel et al., 2011), and women as submissive (Gerber, 1991), alongside the “domestic violence stereotype” of dominant, aggressive men violently abusing weak, vulnerable women (Dutton & White, 2013), may influence the ability of participants to correctly identify and label both parties as both victim and perpetrator, or as primarily perpetrating and being victimized by abuse. Interestingly, the patterns described above were not reflected in judgments of responsibility. Specifically, even though participants found men and women to be less responsible in scenarios where there opposite sex had initiated the abuse, this did not appear strong enough to influence their labelling of men as victims, or women as perpetrators.

Such attitudes may also have been reflected in participants’ judgments of seriousness, as a borderline significant result suggested that participants judged female-dominated scenarios as less serious than either male dominated or scenarios where there were similar levels of aggression from both. Such results speak to previous research that sees women’s violence judged as less severe (e.g., Sorenson & Taylor, 2005), and less likely to be condemned in comparison with men’s aggression (e.g., Felson & Feld, 2009). These findings also reflect patterns of judgments given in response to unidirectional scenarios, where male-perpetrated violence is judged as more serious than that perpetrated by women (e.g., Hine, 2019; Poorman et al., 2003; E. P. Seelau et al., 2003; S. M. Seelau & Seelau, 2005). Furthermore, participants in this study were less likely to suggest that the man call the police, even in female-dominated scenarios (although this was only approaching significance). This compliments research utilizing unidirectional abuse scenarios regarding recommendations to invoke support from law enforcement in male-perpetrated scenarios (Felson & Feld, 2009).

Considering the proportion of abuse that can be classified as bidirectional (Langhinrichsen-Rohling, Misra, et al., 2012), while it is encouraging to see that a significant proportion of participants did choose the option of "both", it is also concerning that participants so infrequently utilized the labels of “perpetrator” and “victim” for women and men respectively, especially when the proportion of abuse would suggest this to be the case. Indeed, it is worth highlighting that, across all scenarios, both the men and women involved are technically victims *and* perpetrators, just to varying degrees. And yet, when participants did assign a primary victim/perpetrator, they did so in line with stereotypes, and not the information before them. Results thus raise specific implications for men experiencing IPV. For example, men are generally less likely to seek help than women (Addis & Mahalik, 2003), an effect exacerbated by a male gender-role dictating stoicism and self-reliance (e.g., Vogel et al., 2011). Indeed, many men report feeling shame and/or embarrassment following victimization at not meeting gender-role expectations (Hogan, 2016) dictating that they should be strong and cope on their own (Bates, 2019b). This surely also contributes to men’s ability to identify as a “victim” in the first place (Machado et al., 2016), and thus help-seek. Research supports this notion, with men reporting that the most significant barrier to help-seeking is the fear of not being taken seriously, or not being believed (Drijber et al., 2013). Social support systems and services have also been shown to be reluctant in acknowledging or recognizing men’s victimization (Tsui, 2014; Tsui et al., 2010), and this is reflected in the accounts of men who discuss their further victimization by services after being laughed at, blamed for their victimization, or not believed because of their physicality (Bates, 2019b). Research further suggests that the prejudices and stereotypes that exist within the IPV service system leaves men vulnerable to legal and administrative aggression (Tilbrook et al., 2010), where by a partner manipulates services and systems at the expense of the other (Bates, 2019a, 2019c; Hines et al., 2007). Considering that effective service responses are often key in helping victims leave an abusive relationship (Waldrop & Resick, 2004), it is essential that damaging narratives and attitudes do not affect provision and that men are treated without prejudice. However, the results of the current study, as well as those seen in the previous literature, indicate that these stereotypes permeate the wider societal discourse around IPV, which may indirectly leave many men vulnerable and without support.

This study should be considered a pilot study in the development of our understanding about perceptions of bidirectional IPV, with a relatively small and unrepresentative sample size yielding enough statistical power to produce significant differences and demonstrating the magnitude of the underlying effects. That being said, there were several limitations to this study, most of which concern the accurate conceptualization of abuse. Specifically, while the novelty of this study lies in the presentation of bidirectional abuse, no direct comparison with unidirectional abuse (e.g., for judgments of seriousness) is included. This would provide important insight into the manifestation and influence of abuse stereotypes within different contexts, and the associated implications for individuals involved. Moreover, this study still only presents a one-time incident of abuse (a limitation highlighted in previous research, Hine, 2019). In reality, domestic violence is often much more complicated, involving a pattern of behavior over time, and the use of control and coercive practices (Langhinrichsen-Rohling, Misra, et al., 2012). This is further exacerbated by complicated evaluations regarding provocation, blame, and responsibility that are evoked in response to bidirectional abuse, and that coalesce with beliefs regarding gender stereotypes, and theoretical models of IPV. For example, questioning whether one act of aggression by one partner, in response to years of abuse at the hands of the other, is enough to categorize the abuse as mutually perpetrated. Such considerations are particularly pertinent when considering the discourse around female aggression as solely motivated by self-defense (Dutton & Corvo, 2006), and several high profile cases centered around the subject (e.g., the case of Sally Challen in the United Kingdom). Future research should therefore seek to utilize examples of IPV that are as representative of experiential accounts as possible, and explore the manifestations of gender norms therein. Other smaller limitations, such as the lack of information regarding participants’ degree program (and subsequent evaluation of the potential impact of different courses on judgments of IPV), should also be noted.

Regardless, results from this study still provide an important insight into the labeling and judgment of “victims” and “perpetrators” within bidirectional IPV scenarios. They primparily suggest that significant issues present for individuals experiencing mutual IPV who do not “fit” with stereotypes regarding “typical” victims and perpetrators, or “typical” gender norms and roles (in a similar way to those experiencing unidirectional abuse). Such preliminary findings should thus stimulate further research on the conceptualization of bidirectional abuse in the minds of the general public, service providers, and those involved. Moreover, exploration of the use and necessity of binary victim/perpetrator labels, and the influence of language itself on the experiences of male victims, is particularly needed.
